# Tubular Cardiac Tissues Derived from Human Induced Pluripotent Stem Cells Generate Pulse Pressure *In Vivo*

**DOI:** 10.1038/srep45499

**Published:** 2017-03-30

**Authors:** Hiroyoshi Seta, Katsuhisa Matsuura, Hidekazu Sekine, Kenji Yamazaki, Tatsuya Shimizu

**Affiliations:** 1 Department of Cardiovascular Surgery, Tokyo Women’s Medical University, 8-1 Kawada-cho, Shinjuku, Tokyo 162-8666, Japan; 2 Institute of Advanced Biomedical Engineering and Science, Tokyo Women’s Medical University, 8-1 Kawada-cho, Shinjuku, Tokyo 162-8666, Japan; 3 Department of Cardiology, Tokyo Women’s Medical University, 8-1 Kawada-cho, Shinjuku, Tokyo 162-8666, Japan

## Abstract

Human induced pluripotent stem (iPS) cell-derived cardiac cells provide the possibility to fabricate cardiac tissues for transplantation. However, it remains unclear human bioengineered cardiac tissues function as a functional pump *in vivo*. Human iPS cells induced to cardiomyocytes in suspension were cultured on temperature-responsive dishes to fabricate cardiac cell sheets. Two pairs of triple-layered sheets were transplanted to wrap around the inferior vena cava (IVC) of nude rats. At 4 weeks after transplantation, inner pressure changes in the IVC were synchronized with electrical activations of the graft. Under 80 pulses per minute electrical stimulation, the inner pressure changes at 8 weeks increased to 9.1 ± 3.2 mmHg, which were accompanied by increases in the baseline inner pressure of the IVC. Immunohistochemical analysis revealed that 0.5-mm-thick cardiac troponin T-positive cardiac tissues, which contained abundant human mitochondria, were clearly engrafted lamellar around the IVC and surrounded by von Willebrand factor-positive capillary vessels. The mRNA expression of several contractile proteins in cardiac tissues at 8 weeks *in vivo* was significantly upregulated compared with those at 4 weeks. We succeeded in generating pulse pressure by tubular human cardiac tissues *in vivo*. This technology might lead to the development of a bioengineered heart assist pump.

Regenerative medicine is expected to be a promising strategy for patients with impaired tissue/organ functions. The integration of stem cell biology with tissue engineering technologies, including cell sheet technology, has already realized regenerative therapy in some areas^1^,^2^,^3^. Current cell therapy using somatic stem cells has improved cardiac functions through mainly paracrine effects such as promotion of angiogenesis. Moreover, the recent advancement of reprogramming technologies^4^ has enabled fabrication of human cardiac tissues *in vitro*^5^ and *in vivo*^6^, which may lead to the development of pulsatile tissues to support circulation.

The development of a circulatory support apparatus is a major challenge to compensate for impaired circulation due to severe heart diseases. Left ventricular assist devices are widely used for patients with severe heart failure due to various ventricular dysfunction, which have decreased the mortality^7^. Circulatory support is also necessary for patients with pulmonary hypertension owing to univentricular heart disease. However, some issues mainly due to the usage of artificial materials including infection, thrombosis and bleeding remain unresolved. Therefore, the development of pulsatile bioengineered tissues capable of assisting systemic and pulmonary circulation may provide us with some cues. We previously reported that tubular tissues fabricated using cell sheets of neonatal rat cardiomyocytes exhibited measurable inner pressure changes evoked by tube contraction *in vitro*^8^. Furthermore, transplantation of rat cardiac cell sheets around the rat abdominal aorta led to inner pressure changes of about 6 mmHg according to their contraction when the myocardial tubes were clamped to distinguish the graft-mediated pulse pressure changes from host pulse pressure^9^. The transplanted cardiac tissues around aorta are exposed to the stretch owing to host pulse pressure, which might lead to promote cardiomyocyte maturation and better function. Conversely, the transplanted cardiac tissues around venous system, the possible case for patients with Fontan operation against univentricular heart disease, are not exposed to the stretch owing to pulse pressure. Therefore, it is necessary to elucidate the function and maturation status of bioengineered pulsatile human tubular cardiac tissues transplanted around venous system. Furthermore, it remains unclear whether bioengineered tubular cardiac tissues have some fundamental properties as a pulsatile pump such as an impulse-conduction system and contraction responses against pressure load *in vivo*.

In the present study, we fabricated human tubular cardiac tissues derived from induced pluripotent stem (iPS) cells around the rat inferior vena cava (IVC). Cell sheet-based human tubular cardiac tissues exhibited inner pressure changes according to their pulsation when transplanted around the rat IVC. Transplanted cardiac tissues were engrafted and matured over time *in vivo*.

## Results

### Characteristics of human cardiac cell sheets

**Figure 1:**
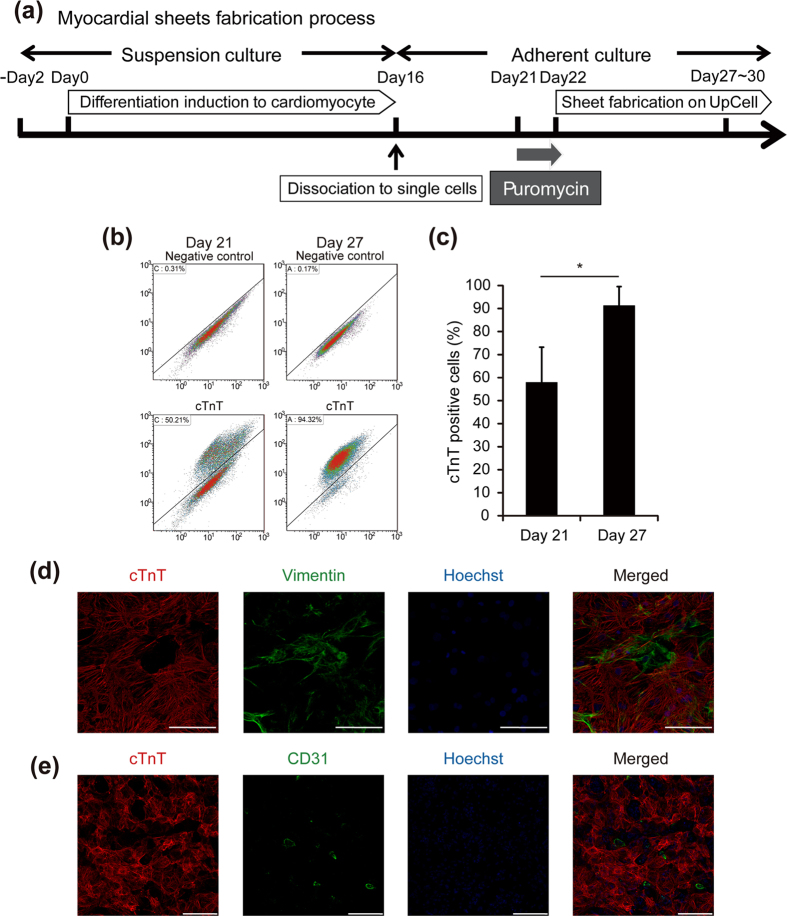
Preparation of human cardiac cell sheets. (**a**) Schematic illustration of the culture process including cardiac differentiation, cardiomyocyte purification, and cell sheet fabrication. (**b,c**) Flow cytometric analysis of cells on days 21 and 27 of differentiation. (**b**) Representative images of flow cytometry analysis. (**c**) The percentage of cardiac troponin T (cTnT)-positive cells was calculated and shown in the graph (n = 5). *p < 0.05. (**d,e**) High content confocal image analysis of cells in cardiac cell sheets (day 27). Nuclei were stained with Hoechst 33258. (**d**) Vimentin (green) expression in cTnT-positive cells (red). Bars, 100 μm. (**e**) CD31 (green) expression in cTnT-positive cells (red). Bars, 200 μm. All data are represented as means ± SD.

First, we attempted to enrich cardiomyocytes in cell sheets to achieve the functional outcomes and prevent tumor formation after transplantation. Using human iPS cells that expressed a puromycin-resistance gene under the control of the mouse α-myosin heavy chain (α-MHC) promoter, puromycin treatment after cardiac differentiation significantly increased the percentage of cardiomyocytes from 57.9 ± 15.3% to 91.2 ± 8.3% (Fig. 1a–c). Cardiac cell sheets were mainly composed of cardiomyocytes and partially non-cardiomyocytes including vimentin-positive fibroblasts and CD31-positive endothelial cells (Fig. 1d,e).

### Transplantation of cardiac cell sheets around the IVC

**Figure 2:**
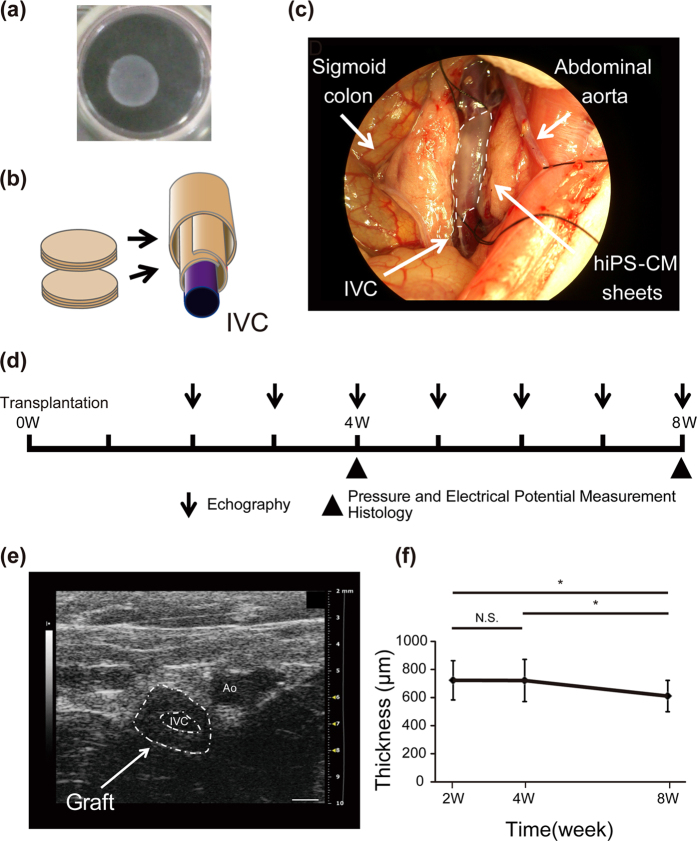
Transplantation of cardiac cell sheets around the inferior vena cava (IVC). (**a**) Representative macroscopic image of a monolayer cell sheet. (**b**) Schematic illustration of transplantation of two pairs of triple-layered cardiac cell sheets around the IVC. (**c**) Macroscopic image of cardiac cell sheet transplantation around the IVC. The area surrounded by white dots indicates the transplanted cardiac cell sheets around the IVC. (**d**) Schematic illustration of the time course of transplantation and analysis. (**e**) Representative image of echographic analysis at 4 weeks after transplantation. The area surrounded by white dots indicates the transplanted tissues around the IVC. Ao: Aorta. Bars, 1 mm. (**f**) Echographic analysis of the transplanted tissue thickness 2–8 weeks after transplantation (n = 4). *p < 0.05. All data are represented as means ± SD.

On days 27–30 of cardiac induction, monolayer cardiac cell sheets were detached by lowering the culture temperature (Fig. 2a). To fabricate tubular cardiac tissues, we created two pairs of triple-layered cardiac cell sheets *in vitro* and transplanted these cell sheets by wrapping them around the IVC of nude rats (Fig. 2b–d). At 2 weeks after transplantation, a low density area around the IVC in 10 of 13 transplanted rats was clearly observed by echography. The thickness of this area was maintained over 600 μm until 8 weeks (2 weeks: 723 ± 140 μm, 4 weeks: 721 ± 150 μm, 8 weeks: 610 ± 111 μm, 2 weeks vs 4 weeks: *p* = 0.97. 2 weeks vs 8 weeks: *p* < 0.05. 4 weeks vs 8 weeks: *p* < 0.05.) (Fig. 2e,f). Spontaneous beating of transplanted cardiac tissues was also observed from 2 or 3 weeks after transplantation by echography (Supplementary Video 1, 2) and macroscopically (Supplementary Video 3) in all rats which showed a low density area around the IVC. The beating was clearly independent of host artery pulsation and maintained at least until 8 weeks.

**Figure 3:**
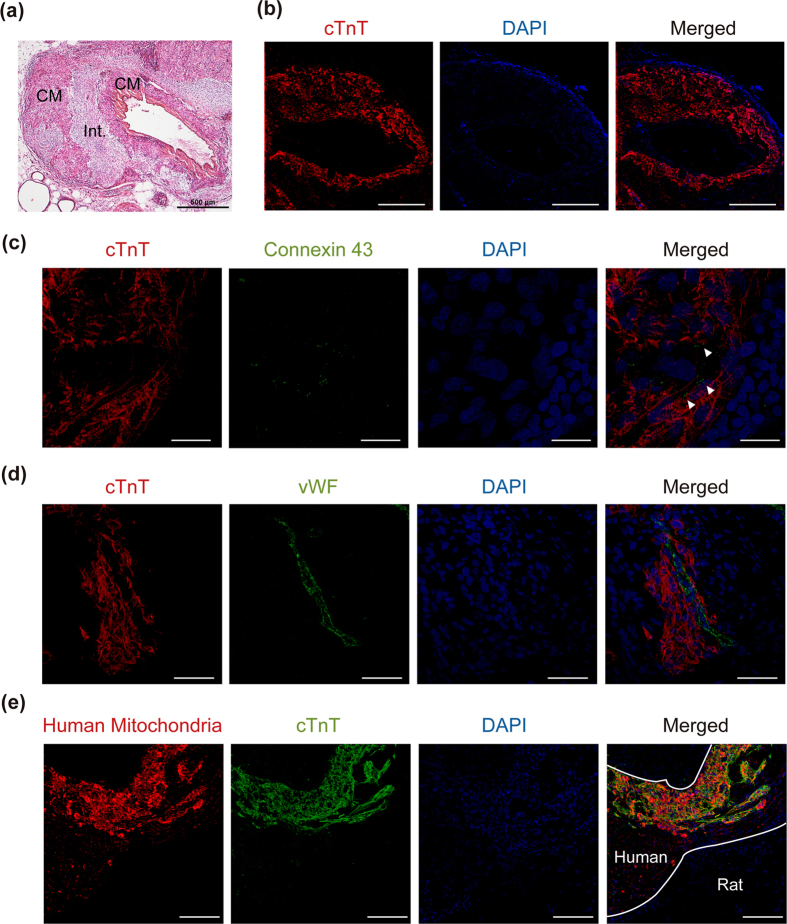
Immunohistochemical analysis of transplanted tissues. (**a**) Hematoxylin-eosin staining of transplanted tissues at 4 weeks. CM and Int. indicate cardiomyocyte and interstitial layer, respectively. Bars, 500 μm. (**b–e**) Immunofluorescence analysis of transplanted tissues around the IVC at 4 weeks. (**b**) Cardiac troponin T (cTnT)-positive cells (red). Nuclei were stained with 4′, 6-diamidino-2-phenylindole (DAPI). Bars, 500 μm. (**c**) Connexin 43 (green, arrow heads) expression in the graft area (red). Nuclei were stained with DAPI. Bars, 20 μm. (**d**) von Willebrand factor (vWF)-positive microvascular networks (green) in cTnT-positive cardiac tissues (red). Nuclei were stained with DAPI. Bars: 50 μm. (**e**) Human mitochondria (red) in transplanted cardiac tissues (green). Area between the white lines indicates the transplanted tissues. Nuclei were stained with DAPI. Bars, 100 μm.

Consistent with the results of echographic analysis, immunohistochemical analysis revealed that the thickness of the transplanted tissues was > 500 μm, and no tumor formation was observed (Fig. 3a). Cardiac troponin T (cTnT)-positive cardiomyocytes were clearly engrafted around the IVC, and connexin 43 expression was observed in the graft area (Fig. 3b,c). Transplanted cardiac tissues contained microvessels expressing von Willebrand factor (vWF) (Fig. 3d) and interstitial cells expressing vimentin and type 1 collagen (Supplementary Fig. 1). As shown in Supplementary Fig. 2, small amount of vWF-positive cells that co-expressed human mitochondria were observed. However, many of vWF-positive cells were negative for human mitochondria. These findings suggest that CD31-positive endothelial cells in cardiac cell sheets *in vitro* might slightly contribute to the microvascular networks in graft tissues, but the microvascular networks in the graft tissues are mainly derived from host rat. Because it is well known that cardiomyocytes contain many mitochondria, human mitochondria were abundant in cTnT-positive cardiomyocytes and less present in cTnT-negative interstitial cells (Fig. 3e). These results suggested that human iPS cell-derived cardiac cell sheets were engrafted around the IVC.

### Physiological functions of pulsatile tubular tissues

**Figure 4:**
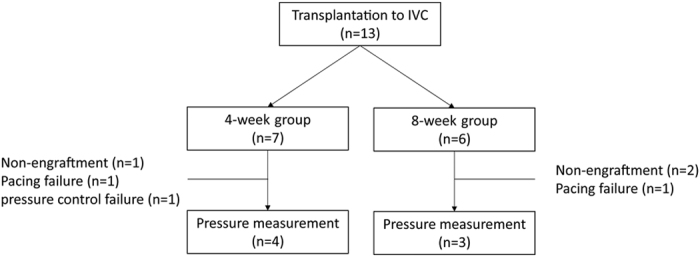
Flow diagram of study population.

**Figure 5:**
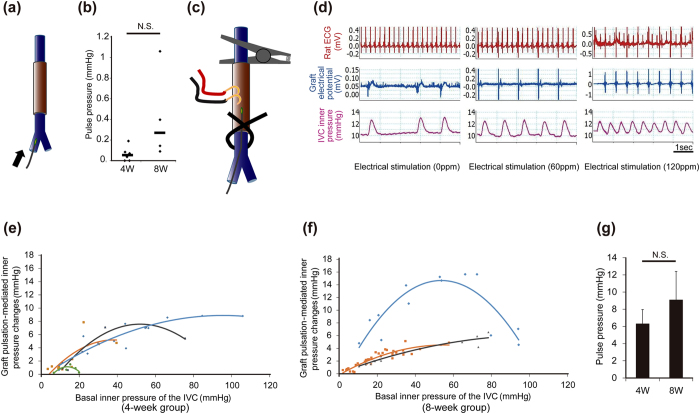
Physiological analysis of tubular cardiac tissues around the IVC. At 4 and 8 weeks after transplantation, rats were intubated and attached to an artificial ventilator under anesthesia. The pressure catheter was inserted into the right femoral vein and the head of the catheter was positioned just into the transplanted cardiac tissue. Pressure measurements were performed under the apnea by turning off the artificial ventilator. (**a,b**) The inner pressure changes evaluation of the transplanted tissue spontaneous pulsation without clamping IVC lumen. (**a**) Schematic illustration of the experiment. (**b**) Pulsation-mediated inner pressure changes before clamping the proximal and distal region of transplanted site in each experiment. (4 weeks, 0–0.19 mmHg, n = 6; 8 weeks, 0.09–1.06 mmHg, n = 4, *p = *0.08). Pressure measurements were performed under spontaneous beating. The data include pacing failure cases and pressure control failure case. Bars indicate the median value. (**c-g**) The inner pressure changes evaluation of the transplanted tissue pulsation with clamping IVC lumen and the electrical stimulation. (**c**) Schematic illustration of the experiment on the clamping site and the electrical stimulation. In order to change basal inner pressure of IVC, the proximal side of IVC was tightly clamped with an atraumatic bulldog clamp and the distal side of IVC was ligated with 6–0 silk with varying degree. (**d**) Representative images of the rat electrocardiogram (ECG) (upper), graft electrical potential (middle), and IVC inner pressure (lower) at 4 weeks. The data under the electrical stimulation at 0 (left), 60 (middle) and 120 (right) pulses per minute (ppm). (**e,f**) Relationship between graft pulsation-mediated inner pressure changes and basal inner pressure of the IVC after clamping. Pressure measurements were performed under electrical stimulation at 80 ppm. (**e**) 4 weeks (n = 4). (**f**) 8 weeks (n = 3). (**g**) Maximum graft pulsation-mediated inner pressure changes in each experiment were calculated and shown in the graph (4 weeks, n = 4; 8 weeks, n = 3, *p = *0.44). All data are represented as means ± SEM.

We examined the physiological functions of transplanted cardiac tissues (Fig. 4). Consistent with the observation that transplanted tissues showed spontaneous beatings, graft tissue-derived electric potentials, which were independent of host heart-derived electric potentials, were observed. The spontaneous graft pulse rate was 54 ± 6 pulses per minute (ppm) at 4 weeks (47–60, n = 6) and 58 ± 22 ppm at 8 weeks (30–78, n = 4), respectively. When we attempted to record the inner pressure changes in the IVC according to graft spontaneous pulsation, slight inner pressure changes were observed at both 4 weeks (4 of 6 rats (0–0.19 mmHg, median: 0.05 mmHg), n = 6) and 8 weeks (4 of 4 rats (0.09–1.06 mmHg, median: 0.27 mmHg), n = 4) (Fig. 5a,b). There was no significant difference in inner pressure changes between 4 weeks and 8 weeks (*p* = 0.08). However, when the basal pressure of the IVC was increased to around 10 mmHg by clamping the proximal and distal region of the IVC to close lumen, the changes of inner pressure were clearly observed (Fig. 5c,d). Graft pulsation followed electrical stimulation until 120 ppm. Subsequently, the relationship between the inner pressure changes and basal inner pressure in the IVC was elucidated at 4 and 8 weeks after transplantation under 80 ppm electrical stimulation. Interestingly, when the basal inner pressure levels of the IVC were increased, graft pulsation-mediated inner pressure changes were also increased accordingly to a certain extent, but the excess increase in the basal inner pressure levels attenuated the graft pulsation-mediated inner pressure changes (Fig. 5e,f). There was no significant difference in the maximum inner pressure changes in clamping conditions between 4 weeks (6.3 ± 1.6 mmHg, n = 4) and 8 weeks (9.1 ± 3.2 mmHg, n = 3) (*p* = 0.44) (Fig. 5g).

### Cardiomyocyte maturation *in vivo*

**Figure 6:**
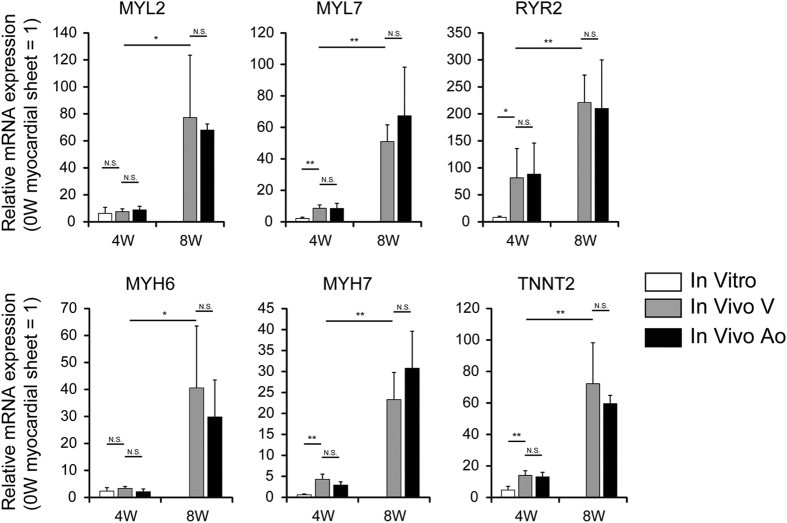
Quantitative polymerase chain reaction analysis of cardiac gene expression in cardiac tissues. Y-axis indicates relative gene expression compared with the cardiac cell sheet before transplantation. *In vitro*, cardiac cells from the same preparation of cell sheets were cultured for a further 4 weeks *in vitro* (n = 4). *In vivo* V, cardiac tissues around the IVC (n = 3). *In vivo* Ao, cardiac tissues around the abdominal aorta (n = 3). All data are represented as means ± SD. *p < 0.05; **p < 0.01.

Our previous report showed that the *in vivo* environment promoted the maturation of iPS cell-derived from cardiomyocytes compared with *in vitro* conditions in terms of myofibril density and the sarcoplasmic reticulum in electron microscopic analysis^6^. Consistent with these observations, the mRNA expression of contractile proteins, such as MYL7, MYH7, TNNT2, and RYR2, a ryanodine receptor, was significantly upregulated at 4 weeks *in vivo* compared with that in cardiac tissues cultured for 4 weeks *in vitro* (Fig. 6). The expression of MYL2 and MYH6 together with MYL7, MYH7, TNNT2 and RYR2 was remarkably upregulated in cardiac tissues at 8 weeks *in vivo* compared with cardiac tissues at 4 weeks *in vivo*.

Because continuous stretching has been reported to induce maturation of human pluripotent stem cell-derived cardiomyocytes^10^, we next examined the contribution of continuous stretching on cardiomyocyte maturation by comparing the cardiac gene expression between cardiac tissues transplanted around the IVC and abdominal aorta. In both case, the cardiomyocytes in the transplanted cardiac tissues were exposed to continuous active stretching based on their own beating rate, while the cardiac tissues around the abdominal aorta would be exposed to more frequent and passive stretching based on host heart-derived pulsation (Supplementary Video 4, Supplementary Fig. 3). However, the mRNA expression of cardiac contractile proteins as well as RYR2 in cardiac tissues around the aorta was comparable to that in cardiac tissues around the IVC.

## Discussion

The present study demonstrated that human cardiac tissues transplanted around the IVC showed physiological inner pressure changes according to their pulsation. Transplanted cardiac tissues were engrafted at least until 8 weeks and well matured *in vivo*. This model will lead to the development of bioengineered human heart assist pump in future clinical situation.

The replacement of injured and fibrotic tissues, due to mainly myocardial infarction, with bioengineered pulsatile cardiac tissues is expected to be useful to ameliorate cardiac dysfunction and subsequently improves QOL and decreases the mortality of patients. However, it remains unclear what extent the bioengineered cardiac tissue contraction directly contribute to heart contraction when in transplantation. Recent reports has suggested that the bioengineered human embryonic stem cell/iPS cell-derived cardiac tissues using fibrin and collagen showed tensile strength around 0.1–11.8 mN/mm ^2^ *in vitro*^11^,^12^. However, because it has been reported that papillary muscle from human left ventricle shows around 40–50 mN/mm ^2^ of contractile force^13^, further development to promote cardiomyocyte maturation will be necessary to sufficiently support the injured left ventricle upon transplantation of bioengineered cardiac tissues in terms of tensile strength. On the other hand, the pressure changes are one of the important parameters to estimate the function of bioengineered cardiac tissues and the development of bioengineered pump will be applicable to support systemic circulation in heart failure patients. In the present study, tubular cardiac tissues around IVC demonstrated not only pulsation-mediated inner pressure changes *in vivo*, but also pressure changes that were comparable to a fundamental property of heart tissues to response to pressure load accompanied with an impulse-conducting system. They produced 9.1 mmHg of inner pressure changes at most at 8 weeks after transplantation. Although that maximum inner pressure change is not fully sufficient to support systemic circulation, it might be applicable to support pulmonary circulation. In other words, tubular cardiac tissues could be applied to the Fontan conduit to support Fontan circulation. Because blood flow from the IVC to pulmonary artery is regulated by only central venous pressure in Fontan circulation, it is difficult for patients with pulmonary hypertension to be treated by the Fontan operation. Although further development of tissue fabrication by cell sheet transplantation on conduits with valves that enable to assist unidirectional blood flow is necessary, tubular cardiac tissues will be able to function as a pulmonary ventricle.

It is well known that pluripotent stem cell-derived cardiomyocytes have immature fetal phenotypes. iPS cell-derived cardiomyocytes have been reported to become mature to some extent over time *in vitro* and *in vivo*^14^. In the present study, the mRNA expression of several cardiac contractile genes and a ryanodine receptor in transplanted cardiac tissues was significantly upregulated over time *in vivo* compared with that in cardiomyocytes *in vitro*. Because the ryanodine receptor is indispensable for calcium-induced calcium release^15^, upregulation of the ryanodine receptor might strengthen the function of bioengineered cardiac tissues. Moreover, we compared the expression of cardiac genes in tissues transplanted around the IVC and abdominal aorta. Because chronic stretch conditions have been reported to induce cardiomyocyte maturation^10^, cardiac gene expression was expected to be upregulated in cardiac tissues around the aorta compared with those around the IVC. However, as shown in Fig. 6, there were no differences between the cardiac gene expression of cardiac tissues around the IVC and aorta. Because the rat heart rate was around 300–400 beats per minute, the transplanted tissues around the aorta were supposed to be exposed to a high stretch frequency. Human iPS cell-derived cardiomyocytes might not tolerant excess rapid pulsation. Therefore, the aorta pulsation might not be effective for maturation of transplanted human cardiac tissues.

There are some limitations in this study. Although cardiac tissues pulsation around the IVC generated inner pressure changes notably, they were recorded in a closed environment and not in an unclamped condition. Also, we observed big variations of inner pressure changes among samples (Fig. 5e,f), which might lead to fail to show the statistical differences on the inner pressure changes between 4 and 8 weeks in spite of the significant difference of several cardiac contractile gene expression. We suppose that the difference of the engraftment and thickness of transplanted cardiac tissues among samples (Supplementary Fig. 4) might be one of the reasons for those issues. We transplanted two pairs of triple-layered cardiac cell sheets and used the hemodynamics data on animals in which the graft pulsation was observed in echography. However, the engraftment and tissue thickness in those animals were different. Although we observed CD31-positive endothelial cells in cardiac cell sheets before transplantation, the number of them was quite low and microvascular network derived from CD31-positive endothelial cells was not observed in cardiac cell sheets *in vitro*. The microvascular network in cell sheets before transplantation has been reported to be indispensable for further engraftment and subsequent improvement of cardiac function in rat myocardial infarction model^16^. In addition, as shown in Supplementary Fig. 2, endothelial cells in the transplanted cardiac tissues were mainly derived from host rat, but not human. Therefore, the cardiac tissues might be exposed to the ischemic condition for a while after the transplantation until the sufficient microvasculature was established in the cardiac tissues, which might lead to less engraftment. We previously reported that step-wise cell sheet layering of pre-vascularized cell sheets^17^ on suitable vascular beds enabled fabrication of multi-layered thickened and dense cardiac tissues *in vivo*^18^, *ex vivo*^19^, and *in vitro*^20^ by inducing microvascular network formation in tissues. Because tubular cardiac tissues with approximately 500-μm thickness generated pressure of 9.1 mmHg in a closed environment, fabrication of thicker cardiac tissues through these technologies might enable to generate pressure more efficiently in an unclamped condition. We suppose that the cardiomyocyte disalignment in cardiac cell sheets might be another reason for issues in this study. It is widely known that cardiomyocytes in hearts show well-ordered alignment, which contributes to generate cardiac output efficiently. However, as shown in Fig. 1d and e, the alignment of cardiomyocytes in cell sheets before transplantation was irregular. Recently several groups including us have reported the strategies to promote cell alignment using some micropatterned surfaces^21^,^22^. The application of such technologies will provide us the well aligned cardiac tissues to generate inner pressure changes more efficiently with less variation.

In conclusion, we showed the possibility of human bioengineered cardiac tissues as a pulsatile bioengineered pump *in vivo*, which might lead to the development of a new type of heart assist devise. In addition to methodological advancement to reduce the risk of tumor formation upon transplantation of iPS cell-derived tissues, further development of tissue fabrication by cell sheet transplantation on conduits with valves in large animal models may provide us with a new therapeutic strategy for heart failure.

## Methods

### Antibodies and regents

The following antibodies were used for flow cytometry and immunocytochemistry: isotype IgG1 mouse monoclonal antibody (Dako, Glostrup, Denmark), anti-cTnT mouse monoclonal antibody (Thermo Scientific, Rockford, IL), anti-SM22 rabbit polyclonal antibody (Abcam, Cambridge, UK), anti-α smooth muscle actin (αSMA) rabbit polyclonal antibody (Abcam), anti-CD31 rabbit polyclonal antibody (Neomarkers, Fremont, CA), anti-connexin 43 rabbit polyclonal antibody (Abcam), anti-vWF rabbit polyclonal antibody (Abcam), anti-human mitochondria mouse monoclonal antibody (Merck Millipore, Darmstadt, Germany), anti-collagen 1 rabbit polyclonal antibody (Abcam), and anti-vimentin rabbit polyclonal antibody (Abcam). Secondary antibodies were purchased from Jackson ImmunoResearch Laboratories (West Grove, PA).

### Cardiac differentiation of hiPS cells, cardiomyocyte purification, and cardiac cell sheet preparation

Human iPS cell line 201B7 was purchased from RIKEN (Tsukuba, Japan). iPS cells were maintained in Primate ES Cell Medium (ReproCELL, Yokohama, Japan) supplemented with 5 ng/ml basic fibroblast growth factor (ReproCELL) on mitomycin C-treated mouse embryonic fibroblasts (ReproCELL) at 37 °C in a humidified atmosphere with 5% CO_2_. Cells were passaged as small clumps every 3–4 days using CTK solution (ReproCELL). To purify cardiomyocytes after the cardiac differentiation, we established iPS cell line that expressed the puromycin-resistance gene under the control of the mouse α-MHC promoter and the neomycin-resistance gene under the control of the rex-1 promoter as described previously^23^,^24^. Briefly a lentiviral vector (α-MHC-pure rex-1-neo) containing the puromycin-resistance gene under the control of the mouse α-MHC promoter and the neomycin-resistance gene under the control of the rex-1 promoter was purchased from Addgene (Cambridge, MA) and transduced into human iPS cells. Only undifferentiated cells expressed the recombinant gene induced by G418 sulfate (Thermo Scientific) for cell expansion. Differentiated cardiomyocytes could be purified by puromycin treatment.

The cardiac differentiation protocol in the bioreactor system (ABLE Co., Tokyo, Japan) has been described previously^5^. Briefly, at 2 days after starting the culture in the bioreactor system with mTeSR1, EBs were cultured in StemPro34 medium containing 50 μg/ml ascorbic acid (Sigma-Aldrich, St. Louis, MO), 2 mM L-glutamine (Life Technologies), and 400 μM 1-thioglycerol (Sigma-Aldrich). The cells were then treated with 0.5 ng/ml bone morphogenic protein-4 (R&D systems, Minneapolis, MN) (days 0–1), 10 ng/ml bone morphogenic protein-4 (days 1–4), 5 ng/ml bFGF (days 1–4), 3 ng/ml activin A (R&D Systems) (days 1–4), 4 μM IWR-1 (Wako, Osaka, Japan) (days 4–6), 5 ng/ml vascular endothelial growth factor (R&D Systems) (days 6–16), and 10 ng/ml bFGF (days 6–16). On day 16 of cardiac differentiation, after cells were dissociated with 0.05% trypsin/EDTA, single cells were plated onto dishes in Dulbecco’s modified Eagle’s medium (DMEM; Sigma-Aldrich) supplemented with 10% fetal bovine serum (FBS) at 37 °C in a humidified atmosphere with 5% CO_2_. At 5 days after starting culture on dishes (day 21), cells were treated with puromycin (Thermo Scientific, 1.5 mg/mL) for 1 day. On the following day, remaining cells were dissociated with 0.05% trypsin/EDTA and re-seeded onto 35-mm temperature-responsive dishes (UpCell; CellSeed, Tokyo, Japan) at 2.1 × 10^5^ cells/cm^2^ in DMEM supplemented with 10% FBS until the harvest of cell sheets (from day 22 to days 27–30). On days 27–30, cells were harvested as monolayer cardiac cell sheets by lowering the culture temperature to 20 °C.

### Flow cytometry

Cells on days 21 and 27 of differentiation were dissociated with 0.05% trypsin/EDTA and fixed with 4% paraformaldehyde for 10 minutes. Fixed cells were stained with an isotype IgG1 or anti-cTnT antibodies as described previously^5^. Stained cells were analyzed using a Gallios (Beckman Coulter, Brea, CA) and Kaluza software (Beckman Coulter).

### Immunocytochemistry

Cells cultured on 24-well plates were fixed with 4% paraformaldehyde and stained with antibodies as described previously^5^. Samples were imaged by ImageXpress (Molecular Devices, Sunnyvale, CA) with MetaXpress and AcuityXpress software (Molecular Devices).

### Transplantation procedure

All animal experiments were approved by the Ethics Committee for Animal Experimentation of Tokyo Women’s Medical University and performed according to the “Guide for the care and Use of Laboratory Animals” published by the US National Institutes of Health (NIH publication No. 85–23, revised 2011).

Monolayer cardiac cell sheets detached from the UpCell after culture at 20 °C for 30 minutes were stacked as a triple-layered sheets. The IVC of > 16-week-old nude rats was exposed by the abdominal median approach under 2% isoflurane anesthesia. A two pieces of triple-layered cardiac cell sheets were wrapped around the IVC to fabricate tubular cardiac tissues. The IVC with cardiac cell sheets was returned to the retroperitoneum space by approximating retroperitoneum with 5–0 absorbable sutures. The abdominal cavity was closed with 5–0 nylon sutures. Thirteen rats were transplanted with cardiac cell sheets and assigned 4-week and 8-week groups.

### Echography

From 2 weeks after transplantation, rats were subjected to abdominal echography every week using the Vevo 2100 System (FUJIFILM VisualSonics, Toronto, Canada) under 2% isoflurane anesthesia. The thickness of the transplanted cardiac tissues was measured at the same position in individual rats up to 8 weeks after transplantation.

### Pressure and electrical potential measurements

At 4 and 8 weeks after transplantation, rats were intubated and attached to an artificial ventilator under 2% isoflurane anesthesia. Two electrodes were attached to the right shoulder and left leg of rats to record the host electrocardiogram. The pressure catheter (1.4Fr; Millar, Houston, TX) was inserted into the right femoral vein and the head of the catheter was positioned just into the transplanted cardiac tissue. After the transplantation site was opened, two electrodes fashioned into a fishhook shape were attached to record the electrical potentials of the transplanted cardiac tissue and another two electrodes were attached for electrical stimulation. To change inner pressure, blood flow was cut off by an atraumatic bulldog clamp at the proximal side of the IVC, and the tightness of clenching by 6–0 silk was modulated at the distal side. Pressure measurements were performed under the apnea by turning off the artificial ventilator. After measurements, rats were sacrificed and transplanted cardiac tissues were resected. PowerLab (ADInstruments, New South Wales, Australia) and LabChart 7 (ADInstruments) were used for recording and analysis.

### Histology and immunohistochemical staining

At 4 and 8 weeks after transplantation, rats were sacrificed and transplanted cardiac tissues were removed. Samples were fixed with 4% paraformaldehyde for a few days and routinely processed into 6-μm-thick frozen sections. Hematoxylin-eosin staining was performed according to standard procedures. Immunohistochemical staining was performed with antibodies as described previously^9^. Samples were imaged using an Eclipse E800 optical microscope (Nikon, Tokyo, Japan) or confocal laser scanning microscope (LSM 510; Carl Zeiss, Jena, Germany) with ZEN software (Carl Zeiss).

### Laser microdissection

A triple-layered cardiac cell sheet was wrapped around the IVC and abdominal aorta of 14-week-old nude rats. After the rats were sacrificed, transplanted cardiac tissues were removed and frozen quickly at 4 and 8 weeks after transplantation. Samples were routinely processed into 20-μm-thick frozen sections by a cryostat and fixed with 70% ethanol for 2 minutes. Then, cresyl violet staining was performed. Cardiomyocytes in serial sections were identified by immunofluorescence staining with the anti-cTnT antibody. Then, samples harvested by laser microdissection using a PALM MicroBeam (Carl Zeiss).

### Real-time reverse transcription-polymerase chain reaction (PCR)

Total RNA was isolated using an RNeasy Micro Kit (QIAGEN, Venlo, the Netherlands). cDNA was synthesized using a High Capacity cDNA Reverse Transcription Kit (Applied Biosystems, Stockholm, Sweden) with random hexamer primers. Real-time PCR analysis of each sample was then performed with a StepOne and StepOnePlus RealTime PCR System (Applied Biosystems). TaqMan assays for real-time PCR (Applied Biosystems) are listed in Supplementary Table 1. The average copy number of gene transcripts was normalized to that of glyceraldehyde-3-phosphate dehydrogenase for each sample.

### Statistical analysis

Statistical analysis was performed with the Student’s t-test or paired t-test. For multiple comparisons of mRNA expression changes over time *in vivo*, the Tukey-Kramer test was performed after single-factor analysis of variance. *P < *0.05 was considered significant. Data are expressed as the mean ± standard deviation except the pressure change expressed as the mean ± standard error.

## Additional Information

**How to cite this article:** Seta, H. *et al*. Tubular Cardiac Tissues Derived from Human Induced Pluripotent Stem Cells Generate Pulse Pressure *In Vivo. Sci. Rep.* **7**, 45499; doi: 10.1038/srep45499 (2017).

**Publisher's note:** Springer Nature remains neutral with regard to jurisdictional claims in published maps and institutional affiliations.

## Supplementary Material

Supplementary Video 1

Supplementary Video 2

Supplementary Video 3

Supplementary Video 4

Supplementary Materials
