# Supplementary material for: Tubular Cardiac Tissues Derived from Human Induced Pluripotent Stem Cells Generate Pulse Pressure In Vivo
Source: Sci Rep. 2017 Mar 30;7:45499. doi: 10.1038/srep45499 (PMC5371992; doi:10.1038/srep45499)
Supplement: Supplementary Materials [file srep45499-s5.pdf]

Supplementary Materials

Tubular Cardiac Tissues Derived from Human Induced Pluripotent Stem Cells Generate Pulse Pressure In Vivo

Hiroyoshi Seta [1,2] M.D., Katsuhisa Matsuura [2,3,*] M.D. Ph. D., Hidekazu Sekine [2] Ph. D., Kenji Yamazaki [1] M.D. Ph. D., Tatsuya Shimizu [2] M.D. Ph. D.

[1]Department of Cardiovascular Surgery, Tokyo Women's Medical University, 8-1 Kawada-cho, Shinjuku, Tokyo 162-8666, Japan

[2]Institute of Advanced Biomedical Engineering and Science, Tokyo Women's Medical University, 8-1 Kawada-cho, Shinjuku, Tokyo 162-8666, Japan

[3]Department of Cardiology, Tokyo Women's Medical University, 8-1 Kawada-cho, Shinjuku, Tokyo 162-8666, Japan

Corresponding author:

Katsuhisa Matsuura, M.D., Ph.D.

E-mail: matsuura.katsuhisa@twmu.ac.jp

Supplementary table

Supplementary Table 1

Gene name	Applied Biosystems TaqMan assay ID
GAPDH	Hs00266705_g1
MYH6	Hs01101425_m1
MYH7	Hs01110632_m1
MYL2	Hs00166405_m1
MYL7	Hs01085598_g1
RYR2	Hs00181461_m1
TNNT2	Hs00165960_m1

Supplementary Figures

Supplementary Figure. 1

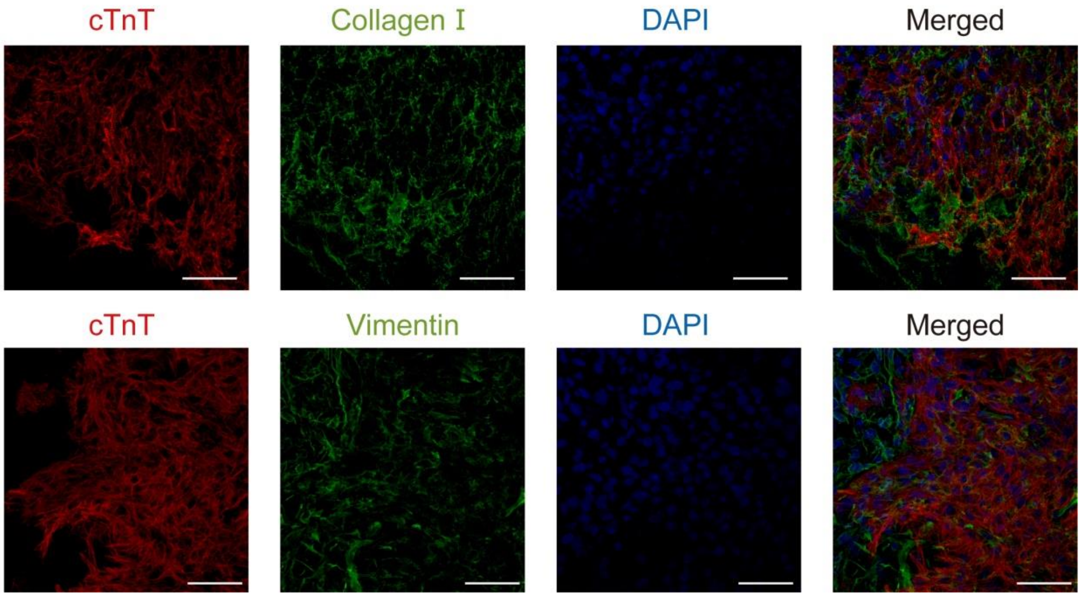

Supplementary Figure. 2

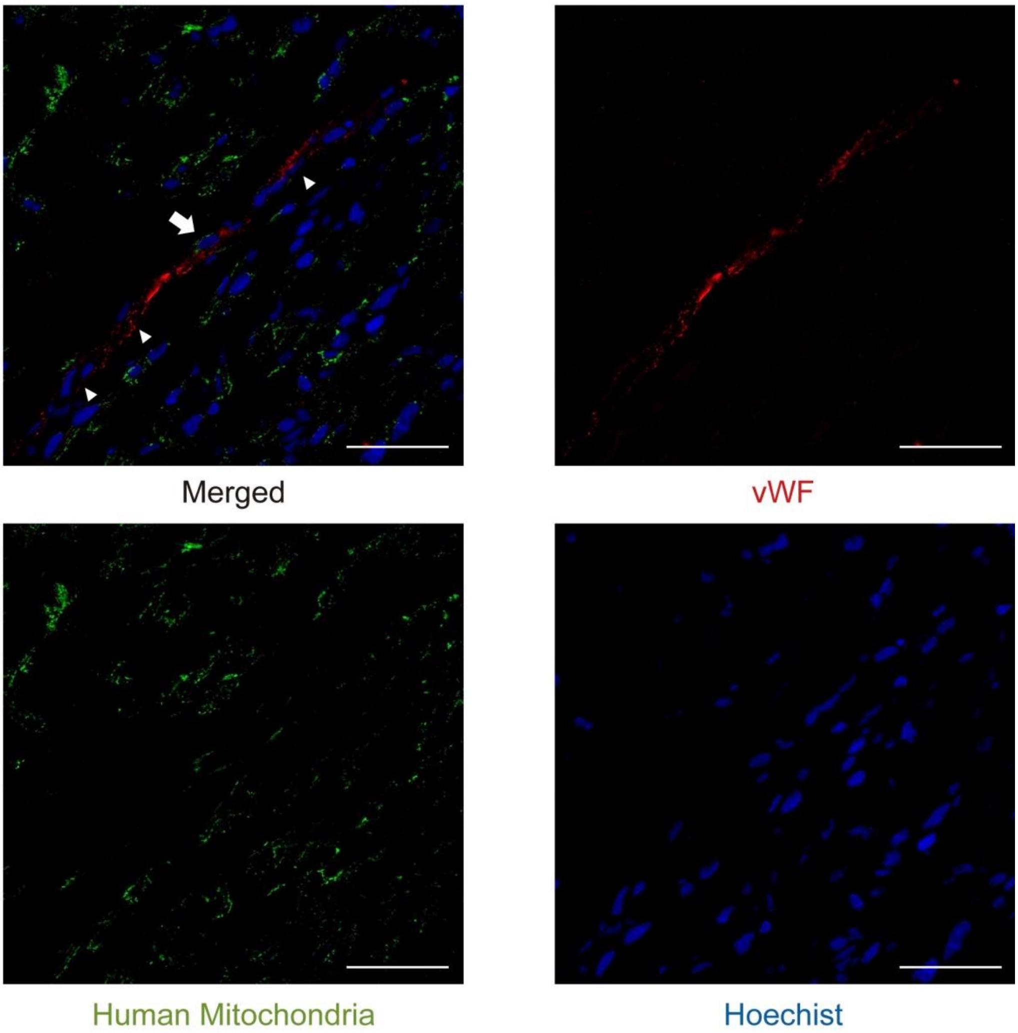

Supplementary Figure. 3

a

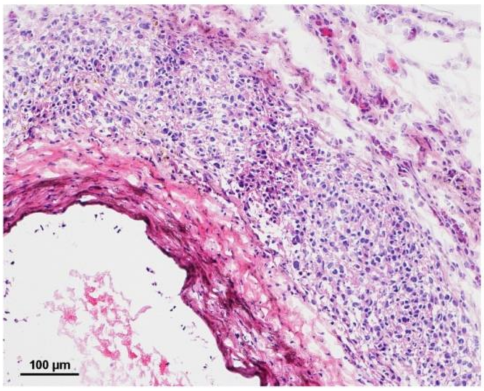

b

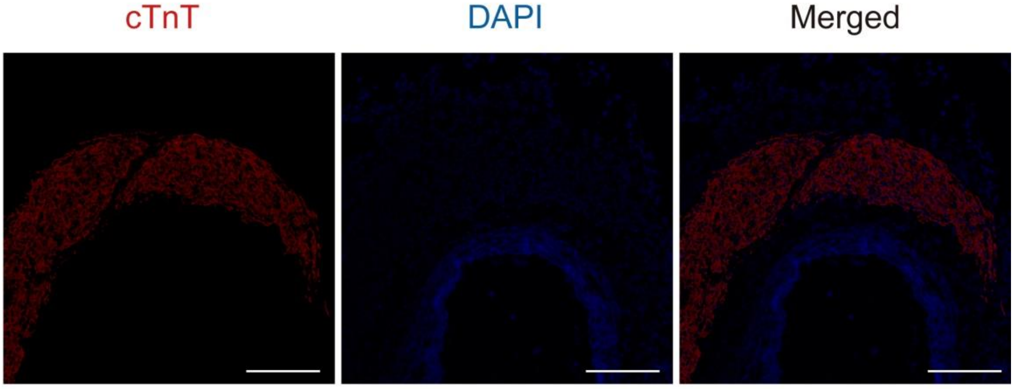

Supplementary Figure. 4

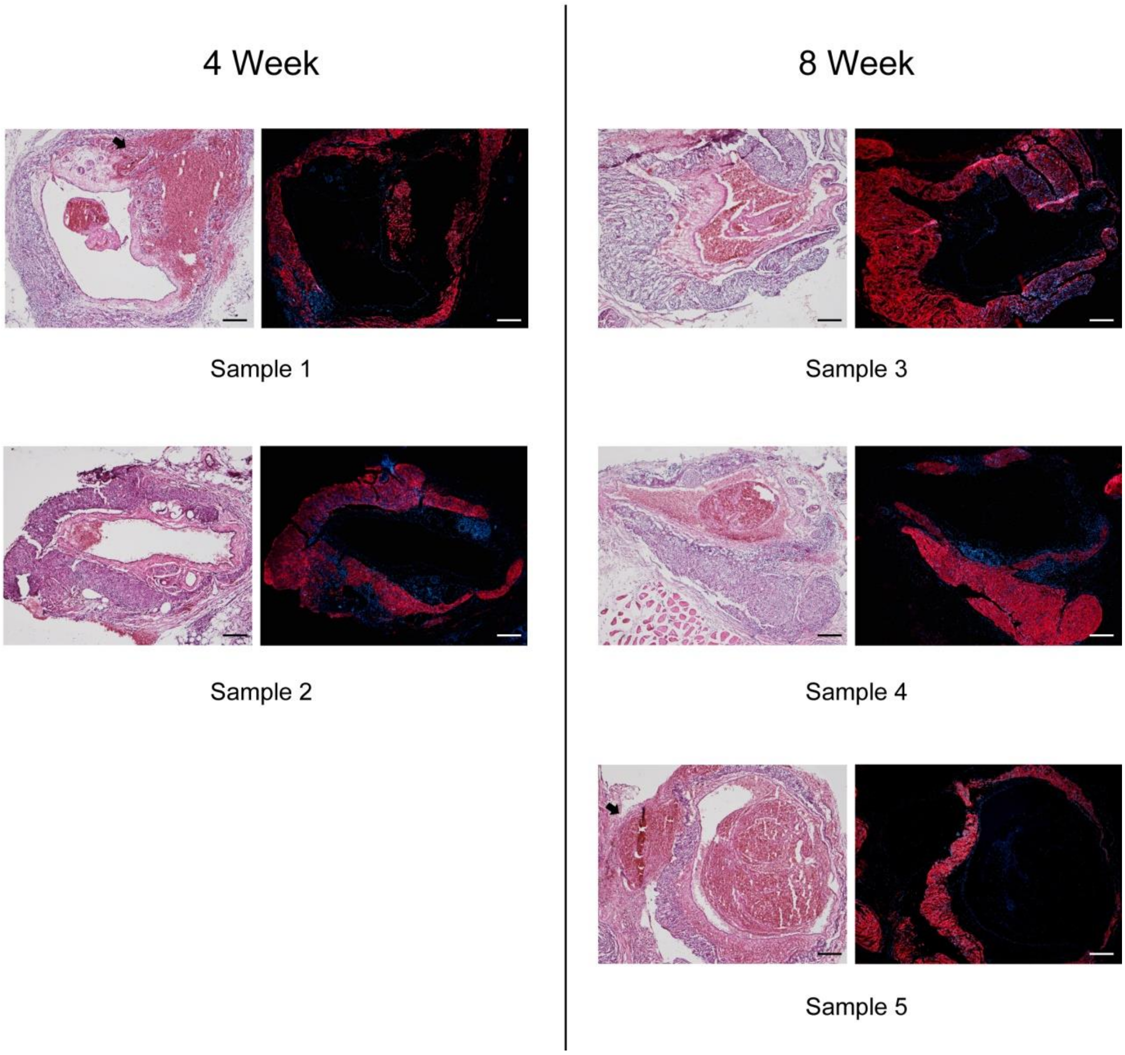

Supplementary figure legends

Supplementary Figure 1. Interstitial cells in cardiac tissues. Immunofluorescence analysis of collagen type 1 (upper, green) and vimentin (lower, green) in cardiac troponin T (cTnT)-positive cardiac tissues (red) at 4 weeks. Nuclei were stained with 4′, 6-diamidino-2-phenylindole (DAPI). Bars, 50 µm.

Supplementary Figure 2. Microvessels in cardiac tissues. Immunofluorescence analysis of von Willebrand factor (red) and human mitochondria (green) in cardiac tissues at 4 weeks. Nuclei were stained with Hoechst 33258. Arrow heads indicate endothelial cells derived from rat and the arrow indicates human mitochondria-positive endothelial cell. Bars, 50 µm.

Supplementary Figure 3. Transplanted cardiac tissue around the abdominal aorta. (a) Hematoxylin-eosin staining of transplanted tissues at 4 weeks. Bars, 100 µm. (b) Immunofluorescence analysis of transplanted cTnT-positive tissues (red) around the abdominal aorta at 4 weeks. Nuclei were stained with DAPI. Bars, 200 µm.

Supplementary Figure 4. Analysis of serial sections of cardiac tissues around the inferior vena

cava at each sample at 4 weeks (left colon) and 8 weeks (right colon) after transplantation. Hematoxylin-eosin staining of transplanted tissues (left). Arrows indicate hematoma due to the puncture of electrode. Immunofluorescence analysis of cTnT-positive tissues (red, right). Nuclei were stained with Hoechst33258. Bars, 200 µm.

Supplementary Video 1. Echographic video of transplanted cardiac tissue around the inferior vena cava at 4 weeks after transplantation.

Supplementary Video 2. Echographic video of sham rat at 1 week after transplantation.

Supplementary Video 3. Macroscopic video of transplanted cardiac tissue after 4 weeks.

Supplementary Video 4. Echographic video of transplanted cardiac tissue around the abdominal aorta at 5 weeks after transplantation.
